# Prediction and Stage Classification of Pressure Ulcers in Intensive Care Patients by Machine Learning

**DOI:** 10.3390/diagnostics15101239

**Published:** 2025-05-14

**Authors:** Mürsel Kahveci, Levent Uğur

**Affiliations:** 1 Anesthesiology and Reanimation, Amasya Training and Reserch Hospital, Amasya University, Amasya 05100, Turkey; drmurselkahveci@yahoo.com; 2 Department of Mechanical Engineering, Faculty of Engineering, Amasya University, Amasya 05100, Turkey

**Keywords:** pressure injury, prediction model, nursing care, machine learning, inpatient

## Abstract

**Background/Objective:** Pressure ulcers are a serious clinical problem associated with high morbidity, mortality and healthcare costs, especially in intensive care unit (ICU) patients. Existing risk assessment tools, such as the Braden Score, are often inadequate in ICU patients and have poor discriminatory power between classes. This increases the need for more sensitive, predictive and integrative systems. The aim of this study was to classify pressure ulcer stages (Stages I–IV) with high accuracy using machine learning algorithms using demographic, clinical and laboratory data of ICU patients and to evaluate the model performance at a level that can be integrated into clinical decision support systems. **Methods:** A total of 200 patients hospitalized in the ICU were included in the study. Using demographic, clinical and laboratory data of the patients, six different machine learning algorithms (SVM, KNN, ANN, Decision Tree, Naive Bayes and Discriminant Analysis) were used for classification. The models were evaluated using confusion matrices, ROC-AUC analyses and metrics such as class-based sensitivity and error rate. **Results:** SVM, KNN and ANN models showed the highest success in classifying pressure ulcer stages, achieving 99% overall accuracy and excellent performance with AUC = 1.00. Variables such as Braden score, albumin and CRP levels contributed significantly to model performance. ROC curves showed that the models provided strong discrimination between classes. Key predictors of pressure ulcer severity included prolonged ICU stay (*p* < 0.001), low albumin (Stage I: 3.4 ± 0.5 g/dL vs. Stage IV: 2.4 ± 0.8 g/dL; *p* < 0.001) and high CRP (Stage I: 28 mg/L vs. Stage IV: 142 mg/L; *p* < 0.001). **Conclusions:** This study shows that machine learning algorithms offer high accuracy and generalization potential in pressure ulcer classification. In particular, the effectiveness of algorithms such as SVM, ANN and KNN in detecting early-stage ulcers is promising in terms of integration into clinical decision support systems. In future studies, the clinical validity of the model should be increased with multicenter datasets and visual-data-based hybrid models.

## 1. Introduction

Patients hospitalized in intensive care units (ICU) constitute a highly vulnerable patient group in terms of pressure ulcers (bedsores) due to the combination of many risk factors such as prolonged immobility, systemic complications and circulatory disorders [1,2,3]. Pressure ulcers are localized tissue damage that develops as a result of prolonged exposure of the skin and underlying tissues to pressure, usually in areas where bony prominences are located. These wounds not only reduce the patient’s quality of life but also lead to serious clinical and economic consequences, such as prolonged hospitalization, development of infection, and increased treatment costs. These wounds in ICU patients play an important role in increasing mortality and morbidity rates [3,4]. The prevalence of pressure ulcers in ICU patients has been reported to range from 10% to 41%; this rate varies depending on the overall health status and quality of care [5].

For the clinical management of pressure ulcers, the staging of these lesions is of great importance. According to the National Pressure Ulcer Advisory Panel (NPUAP), pressure injuries are graded from stage I to stage IV, and this grading is based on the depth of the affected tissue layer of the wound and the extent of damage [6]. Stage I is characterized by persistent erythema or circulatory disturbance, usually occurring without compromised skin integrity. Stage II involves partial loss of epidermis and/or dermis and appears as superficial ulcers. Stage III refers to full-thickness tissue loss and may extend to subcutaneous adipose tissue; necrosis is usually also observed. The most advanced stage, stage IV, describes deep wounds in which all layers of skin and tissue are destroyed and even muscle, tendon or bone tissue are exposed. This staging plays a critical role in both formulating the clinical treatment plan and evaluating the prognosis of wounds [6].

The staging of pressure ulcers plays a critical role not only in determining treatment strategies but also in the early diagnosis of these wounds and prevention of complications. Early diagnosis can directly influence the success of the treatment process and reduce patient morbidity. However, traditional diagnostic methods are often based on visual assessments by medical staff and subjective risk scoring scales such as Braden, Norton or Waterlow. These approaches are inadequate and limited in clinical accuracy, especially in the detection of early-stage ulcers [7]. Therefore, there is an increasing need for more objective and accurate assessment tools in clinical practice.

In this context, machine learning (ML) and artificial intelligence (AI)-based technologies have been applied in healthcare with increasing interest in recent years. ML creates predictive models by learning patterns from large volumes of clinical data and thus makes significant contributions to clinical decision support processes [8,9,10]. In particular, image processing and deep learning techniques have attracted attention with their high accuracy rates in the automatic classification of skin lesions [11]. These technologies enable early diagnosis of pressure ulcers, facilitate their classification into stages, and allow personalization of treatment plans. In light of these developments, it is predicted that machine learning-supported models can replace traditional scoring systems as a more reliable and effective alternative [12,13,14,15,16].

However, many studies in the literature have focused only on predicting the presence of pressure ulcers, and there is a lack of research on the classification of these wounds according to their severity. However, pressure ulcers are clinically divided into various stages and each stage requires different approaches in treatment planning. Such differentiation of wounds may contribute to the personalization of care to be applied to patients and the development of more effective intervention strategies. Therefore, the main objective of this study is to develop a model that can classify these wounds according to their stages while predicting the risk of developing pressure ulcers in patients hospitalized in the intensive care unit through machine learning algorithms. With this approach, we aim to create individualized care strategies and provide a more proactive healthcare service for the prevention of pressure ulcers.

## 2. Materials and Methods

### 2.1. Study Design and Data Collection

This study is a retrospective cohort study conducted in the Intensive Care Unit (ICU) of Amasya University Faculty of Medicine Hospital. For the purpose of the study, the electronic health records of a total of 2500 patients followed up in the ICU with a capacity of 60 beds between 2020 and 2025 were analyzed. Data were obtained through the hospital information management system (HIS) and included age, gender, length of stay, comorbidities (malignancy, diabetes, coronary artery disease, neuropathy), intubation status, vital signs and laboratory parameters, as well as presence and staging of pressure ulcers. Pressure ulcers were staged by in-hospital clinical staff according to the National Pressure Injury Advisory Panel (NPUAP) guidelines and these assessments were integrated into the dataset.

Inclusion criteria were as follows: 18 years of age or older, at least 48 h of ICU stay, complete information in electronic health records and pressure ulcer assessment. Exclusion criteria were as follows: patients with incomplete or inaccurate records during hospitalization, patients with a previous diagnosis of pressure ulcers in another healthcare institution, patients with pressure ulcers detected during ICU admission and individuals with a length of stay of less than 48 h.

**Figure 1:**
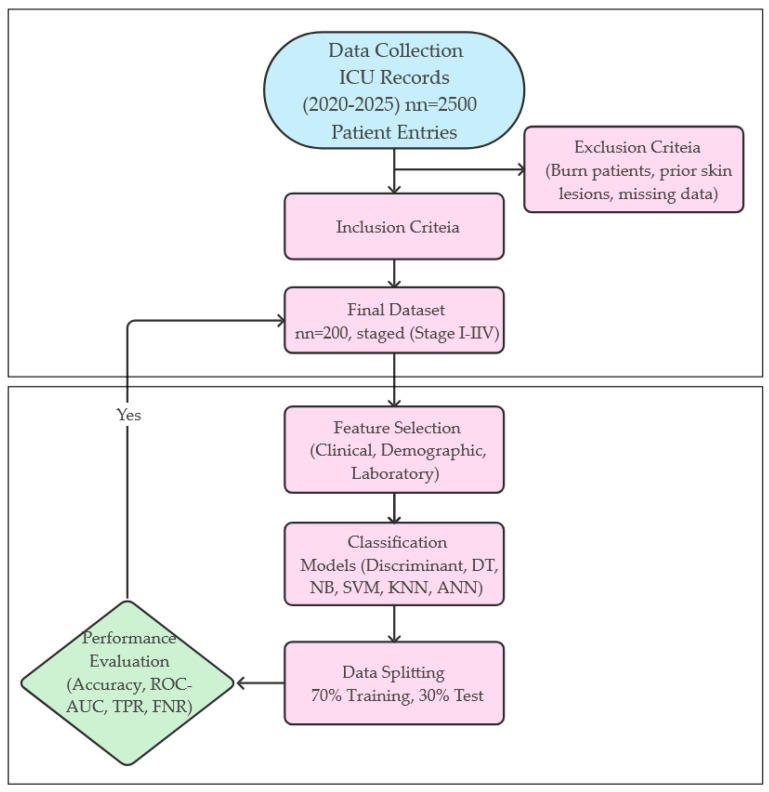
Flowchart.

As a result of the final evaluation, the data of 200 patients who met the criteria were included in the analysis process. Of these patients, 105 were female with a mean age of 67.8 ± 10.1 years and 95 were male with a mean age of 70.2 ± 10.6 years. Ethics committee approval for the study was obtained from Amasya University Ethics Committee (2025/69). Detailed demographic information of the patients is presented in Table 1. The methodological process applied in the study is systematically presented in Figure 1 from data collection to model evaluation. This flowchart summarizes all stages from patient inclusion criteria to modeling and performance measures.

The dataset was divided into three subgroups during the model development process: 70% training, 15% validation and 15% test data. This separation was performed in order to maximize the learning capacity of the model and to increase its validation and generalization power. In addition, a k-fold cross-validation method was applied to assess the statistical reliability of the model. This method helped to prevent overfitting by evaluating the performance of the model on different data subsets.

### 2.2. Clinical Data of the Patients

Special attention was paid to the clinical variables analyzed in the study. These variables played an important role in both assessing the risk of pressure ulcer development and increasing the predictive power of the model by being among the input parameters of the machine learning-based classification model. Clinical data are summarized in Table 2. Mean arterial pressure (MAP) reflects the hemodynamic stability of the patients and its mean value was calculated as 80 ± 12.3 mmHg. The mean heart rate was 88.5 ± 15.2 beats/min, respiratory rate 18.6 ± 4.1 respirations/min and body temperature 36.8 ± 0.7 °C. In addition, 66% (*n* = 132) of the patients were intubated and the mean duration of stay on mechanical ventilator was 13 ± 6.5 days. These parameters were considered as critical indicators representing hemodynamic and respiratory status as well as general health status in intensive care unit patients.

### 2.3. Laboratory Results

Laboratory data include biochemical and gas exchange indicators such as hemoglobin (g/dL), white blood cell (WBC, 10^3^/µL), blood glucose (mg/dL), albumin (g/dL), creatinine (mg/dL), PaO_2_ (mmHg) and pCO_2_ (mmHg). These parameters are important in terms of providing information about the general metabolic status of the patient and tissue oxygenation. The laboratory results of the patients are given in Table 2.

### 2.4. Comorbidities

The patients’ comorbidities were also included in the dataset. These comorbidities include various systemic diseases that have a potential effect on the development of pressure ulcers. The most common cardiovascular system diseases were recorded as hypertension (46%) (*n* = 92), coronary artery disease (42%) (*n* = 84) and heart failure (19%) (*n* = 38). Endocrine system disorders included diabetes mellitus (38%) (*n* = 76) and thyroid dysfunction (12%) (*n* = 24). In the neurological diseases group, there were cerebrovascular events (22.5%) (*n* = 45) and neuropathy (16%) (*n* = 32). Other system diseases included chronic lung disease (20.5%) (*n* = 41), malignancy (14.5%) (*n* = 29) and chronic renal failure (14%) (*n* = 28). These comorbid conditions were evaluated as important factors that could increase the risk of pressure ulcers by affecting the general clinical condition of the patients.

### 2.5. Statistical Analysis

IBM SPSS Statistics 25.0 (IBM Corp., Armonk, NY, USA) software was used to analyze the data. Normality distribution was evaluated by a Shapiro–Wilk test and appropriate statistical methods were selected according to the distribution characteristics of the data. Mean ± standard deviation was used for continuous variables with normal distribution and median values were used for those without normal distribution.

In intergroup comparisons, one-way analysis of variance (ANOVA) was used for normally distributed parametric data and a Kruskal–Wallis H test was used for nonparametric data not normally distributed. In cases where a statistically significant difference was detected in the ANOVA test, in-group comparisons were made using Tukey’s HSD (Honestly Significant Difference) test as a post hoc analysis. Pearson’s chi-squared test was used to compare categorical variables.

To evaluate the effect size, eta-square (η^2^) was calculated for parametric tests and epsilon-square (ε^2^) for nonparametric tests. For multivariate analysis, an ordinal logistic regression model was developed to determine the factors affecting pressure ulcers stages. The statistical significance level was accepted as *p* < 0.05 and all tests were performed two-way.

### 2.6. Classification

In this study, the classification process was performed using comprehensive clinical, demographic and laboratory data from patients hospitalized in the intensive care unit. The aim was to accurately predict the pressure ulcer stages (Stage I–IV) of the patients using this multidimensional dataset. No feature selection was applied in the study; instead, all available variables were directly included in the classification models. This approach ensures that the model utilizes all possible sources of information, while avoiding the exclusion of variables that are potentially important for decision support systems, especially in clinical applications.

The classification models were trained based on supervised learning algorithms. Accordingly, decision trees (DTs), linear discriminant analysis (LDA), Naive Bayes (NB), support vector machines (SVMs), k-Nearest Neighbor (k-NN) and artificial neural networks (ANNs) were used to evaluate the success of the model in predicting pressure ulcer stages. In particular, LDA was used to optimize the intra-class and inter-class variance ratio to increase the discrimination between classes, contributing to class separation rather than dimensionality reduction [17,18]. Each of these methods was tested with 10-fold cross-validation through statistical metrics such as classification accuracy, sensitivity, specificity and F1 score, and the overall success and clinical applicability of the model were interpreted according to these results.

## 3. Results

In this study, we compared the performance of various machine learning algorithms in the classification of pressure ulcer stages. Decision Tree (DT), Naive Bayes (NB), Support Vector Machine (SVM), K-Nearest Neighbor (KNN), Artificial Neural Network (ANN) and Optimizable Discriminant Analysis methods were used. For model evaluation, confusion matrices, true positive rates (TPR), false negative rates (FNR) and overall accuracy (Accuracy) values were considered. All the results obtained are summarized in Table 3.

In terms of classification accuracy, the highest performance was observed in SVM, KNN, ANN and Optimizable Discriminant models. All of these models achieved an overall classification accuracy of 99%. The SVM model achieved 100% accuracy in Stage I, II and III and misclassified two cases as Stage I only in Stage IV (TPR: 96%, FNR: 4%). Similarly, ANN and KNN models had a 96% correct classification rate for Stage IV and 100% accuracy in other classes. The Optimizable Discriminant method achieved 99% accuracy with two incorrect predictions only in Stage IV.

The Naive Bayes model performed well in general, with an accuracy rate of 94.5%. However, the sensitivity dropped to 88% at Stage III, indicating a 12% false negative classification rate. The DT model showed the lowest overall accuracy (91.5%). In this model, the FNR was as high as 14%, especially in Stage III, while some deviations were also observed in other stages (e.g., TPR 88% for Stage II).

Overall, SVM, KNN and ANN algorithms in particular stood out with their high accuracy, low error rate and strong discrimination between classes. It can be said that these models can be effectively used in clinical decision support systems for the prediction of pressure ulcer stages. The obtained confusion matrices, TPR-FNR distributions and class-based evaluations are presented in Table 4.

In addition to the conventional metrics, we also calculated the Matthews Correlation Coefficient (MCC) and Cohen’s Kappa to better assess model robustness in the presence of potential class imbalance. These additional metrics are summarized in Table 5, which further confirms the high consistency and reliability of the leading models.

### ROC Curves and AUC Analysis

In order to evaluate the predictive power of the classification algorithms used in the study, ROC (Receiver Operating Characteristic) curves and AUC (Area Under the Curve) values were calculated for each of them. The ROC curve graphically shows the sensitivity (True Positive Rate) and specificity (False Positive Rate) values of the model at various threshold values, allowing the discrimination of the model between classes to be measured. The AUC value represents the area under the ROC curve and reflects the overall discrimination performance of the model. The closer the AUC value is to 1, the higher the classification success of the model.

According to the results obtained in this context, Optimizable Discriminant, SVM, KNN and ANN models exhibited excellent discrimination with AUC = 1.00. This shows that these models can classify the pressure ulcer stages accurately. The Optimizable Naive Bayes model performed very well, with AUC = 0.99. The Optimizable Tree model, which performed relatively poorly, still showed an acceptably strong discriminative ability with an AUC = 0.98.

**Figure 2:**
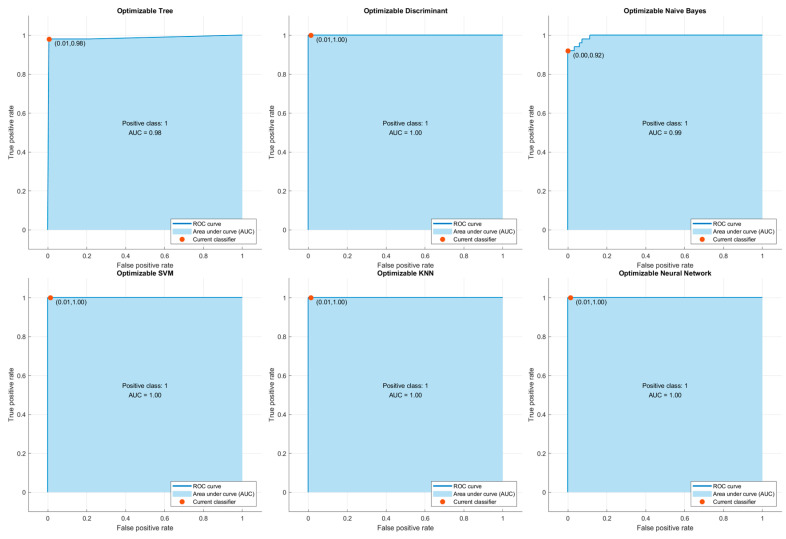
ROC (Receiver Operating Characteristic) curves and AUC (Area Under the Curve) values of six different machine learning algorithms used in the study.

These findings suggest that the AUC scores obtained through the ROC curves strongly support not only the classification accuracy but also the usability of the model in clinical decision support systems. Images of the ROC curves are presented separately in Figure 2.

## 4. Discussion

In this study, the effectiveness of machine learning models developed for the classification of pressure ulcer stages in patients hospitalized in the intensive care unit was evaluated. Six different algorithms were tested, including SVM, ANN, KNN and optimization-based models, and both the class-based accuracy rates and ROC-AUC performances of the models were presented comparatively. According to the findings, SVM, ANN and KNN algorithms especially showed excellent classification performance, with AUC = 1.00. This is a very promising result for clinical decision support systems (CDSSs).

One of the most remarkable findings of our study is that ML models were able to detect early-stage pressure ulcers (Stages I and II) with high accuracy. This is important to overcome the limitations of traditional methods (such as the Braden Scale) in detecting early-stage lesions. This is in line with the study by Xu et al. (2022), who developed a machine learning model for predicting pressure ulcer development in ICU patients and reported that a nomogram combined with the Braden score improved the AUC value up to 0.87 [13]. These results suggest that ML-based systems can provide important support to clinicians in planning both early detection and intervention strategies.

The results of the current study are also consistent with the results of a retrospective cohort analysis by Alderden et al. (2021), in which models using only five easily accessible variables in addition to the Braden scale were as effective as large datasets [12]. Similarly, Cramer et al. (2019) reported that machine learning models provide more successful predictions with EHR data, despite the low predictive value of the Braden score [4]. Similarly, in our study, the Braden score alone did not show a significant discriminatory feature, but clinical parameters such as albumin, CRP and respiratory rate stood out as strong predictors.

Recent studies in the literature clearly demonstrate that machine learning is playing an increasing role in pressure ulcer management [13,19,20,21]. For example, the clinical decision support system (CDSS) developed by Kim et al. (2022) significantly increased nurses’ awareness of pressure ulcer prevention strategies and their ability to recognize risk areas on the skin [22]. In the clinical study conducted by Fergus et al. (2022), an AUC value of 0.92 was achieved in pressure ulcer classification using a mobile-based deep learning system [23]. These results show that machine learning applications can be integrated not only at the theoretical level but also directly into clinical decision-making processes in the field. The high accuracy (99%) and AUC (1.00) values obtained in our current study, especially when combined with the ability to effectively classify early-stage ulcers, reinforce these literature findings and once again confirm the potential of integrating machine learning-based systems into intensive care clinics. In this context, our study both confirms the success of existing technological solutions and reveals that the use of multidimensional clinical data can improve model success.

Our study also supports the two-phase model proposal of Dweekat et al. [24]. In this proposal, a cost-sensitive SVM supported by a genetic algorithm is used to first predict the pressure ulcer evolution and then determine the time at risk by grid search. These approaches open new horizons in resource prioritization and personnel planning.

Another important contribution of our study is comprehensive analysis of the features used in the staging of pressure ulcers. For example, among the laboratory findings, albumin levels (Stage I: 3.4 ± 0.5 g/dL, Stage IV: 2.4 ± 0.8 g/dL; *p* < 0.001) and CRP values (Stage I: 28 mg/L, Stage IV: 142 mg/L; *p* < 0.001) showed a significant correlation with the severity of the wound. These findings are consistent with studies in the literature [25,26]. Cox (2011) reported that low albumin levels and high inflammatory markers are important risk factors for pressure sore development [5]. Similarly, the significant difference in the Braden Score according to stages in our study (Stage I: 16.4 ± 1.7, Stage IV: 7.6 ± 4.0; *p* < 0.001) once again confirms the importance of this score in clinical practice.

### Limitations

Although the study findings demonstrated the effectiveness of machine learning models with high accuracy and AUC values, some limitations should be considered. First, this study was conducted on a homogeneous sample of patients from a single center. This may limit the generalizability of the developed models to different patient profiles and hospital settings. In addition, the dataset mainly consists of structured clinical data, and enriching elements such as visual data were not included in the model performance. In future studies, the integration of imaging data and the use of advanced deep learning techniques such as convolutional neural networks (CNNs) can further improve classification accuracy and predictive capacity. In addition, the fact that the models in the study have not been tested in real-time clinical applications suggests that additional research is needed on the functionality of the algorithms in practice. Despite these limitations, the findings suggest that machine learning-based decision support systems have significant potential in pressure ulcer management and may help to develop more proactive and sensitive care strategies, especially in high-risk patient groups.

## 5. Conclusions

The overall results of this study revealed that machine learning algorithms, especially SVM, KNN and ANN models, can classify pressure ulcer stages in patients monitored in the intensive care unit with high accuracy rates (99%). These algorithms demonstrated superior performance in distinguishing both early ulcers (Stage I and II) and advanced stage lesions (Stage III and IV), offering significant potential in terms of timely intervention and individualized care planning. These findings, supported by ROC-AUC analyses, show that the models are successful not only in terms of overall accuracy but also in terms of class-based sensitivity and selectivity. In addition, the statistically significant relationships between parameters such as albumin level, C-reactive protein (CRP) and Braden score, which are among the basic clinical variables contributing to model performance, and ulcer stage, have once again demonstrated the importance of these variables in risk assessment. The data obtained strongly support machine learning-based classifiers’ ability to be integrated into clinical decision support systems and used as effective and reliable tools. However, the validation of these models in large patient groups, multicenter studies, and with real-time clinical data is critical to increase generalizability and clinical reliability.

## Figures and Tables

**Table 1:** Demographic properties of the patients.

Variable	Stage I (*n* = 50)	Stage II (*n* = 50)	Stage III (*n* = 50)	Stage IV (*n* = 50)	*p*-Value * (All Group Comparison)	Post Hoc
Age (Median ± SD)	68 ± 6.26	71 ± 7.83	74 ± 9.12	73 ± 10.97	<0.001	I-II: 0.012, I-III: <0.001
Gender (F/M)	22/28	24/26	25/25	23/27	0.934	-
Height (cm) (Mean ± SD)	165.1 ± 7.5	166.8 ± 8.2	161.0 ± 9.3	168.3 ± 7.1	0.203	-
Weight (kg) (Mean ± SD)	69.3 ± 10.9	71.7 ± 13.8	64.0 ± 13.1	70.9 ± 12.0	0.287	-
BMI (kg/m^2^) (Mean ± SD)	25.4 ± 3.9	25.8 ± 5.0	24.7 ± 4.5	25.0 ± 4.2	0.638	-

* Statistically significant difference (*p* < 0.05).

**Table 2:** Comparison of laboratory and clinical parameters according to pressure ulcer stages.

Parameter	Stage I (*n* = 50)	Stage II (*n* = 50)	Stage III (*n* = 50)	Stage IV (*n* = 50)	*p* Value	Effect Size (η^2^)	Post Hoc (Significant Groups)
Clinical Parameters							
ICU Length of Stay (days)	7 [5–10]	11 [8–15]	19 [14–26]	34 [25–45]	<0.001	0.38	Between all groups < 0.01
Mechanical Vent. Duration (days)	3 [1–5]	7 [4–10]	16 [10–24]	26 [22–31]	<0.001	0.42	I-II-III-IV chain
Glasgow Coma Score	12 ± 2	10 ± 3	9 ± 3	8 ± 4	<0.001	0.35	I-II, I-III, I-IV
Braden Score	16.4 ± 1.7	13.7 ± 2.5	10.6 ± 3.3	7.6 ± 4.0	<0.001	0.51	All comparisons < 0.001
MAP (mmHg)	84 ± 6	82 ± 7	78 ± 8	76 ± 9	0.012	0.18	I-III, I-IV
Laboratory Results							
Hemoglobin (g/dL)	10.2 ± 1.1	9.4 ± 1.3	8.8 ± 1.5	8.1 ± 1.7	<0.001	0.29	I-II-III-IV chain
Albumin (g/dL)	3.4 ± 0.5	3.0 ± 0.6	2.7 ± 0.7	2.4 ± 0.8	<0.001	0.33	Between all groups < 0.05
CRP (mg/L)	28 [15–42]	56 [35–78]	98 [64–132]	142 [95–185]	<0.001	0.45	I-II-III-IV chain
Leukocytes (10^3^/µL)	9.8 ± 2.1	11.2 ± 3.0	12.5 ± 3.5	13.8 ± 4.0	<0.001	0.27	I-III, I-IV, II-IV
Creatinine (mg/dL)	1.0 ± 0.3	1.1 ± 0.4	1.3 ± 0.5	1.5 ± 0.6	0.001	0.22	I-IV, II-IV
PaO_2_/FiO_2_	285 ± 45	235 ± 52	190 ± 60	150 ± 65	<0.001	0.41	I-II-III-IV chain
WBC (10^3^/µL) (Mean ± SD)	9.8 ± 2.1	11.2 ± 3.0	12.5 ± 3.5	13.8 ± 4.0	<0.001	0.27	I-III, I-IV, II-IV
Blood Lactate (mmol/L) (Mean ± SD)	1.4 ± 0.5	1.8 ± 0.6	2.3 ± 0.7	2.9 ± 0.9	<0.001	0.39	I-III, I-IV, II-IV

**Table 3:** Confusion matrix.

		Predicted Class			
DT [all features]		Stage I	Stage II	Stage III	Stage IV
True class	Stage I	49	1	0	0
	Stage II	1	44	3	2
	Stage III	0	2	43	5
	Stage IV	0	2	1	47
Optimizable Discriminant [all features]		Stage I	Stage II	Stage III	Stage IV
True class	Stage I	50	0	0	0
	Stage II	0	50	0	0
	Stage III	0	0	50	0
	Stage IV	2	0	0	48
NB [all features]		Stage I	Stage II	Stage III	Stage IV
True class	Stage I	46	4	0	0
	Stage II	0	49	1	0
	Stage III	0	6	44	0
	Stage IV	0	0	0	50
SVM [all features]		Stage I	Stage II	Stage III	Stage IV
True class	Stage I	50	0	0	0
	Stage II	0	50	0	0
	Stage III	0	0	50	0
	Stage IV	2	0	0	48
KNN [all features]		Stage I	Stage II	Stage III	Stage IV
True class	Stage I	50	0	0	0
	Stage II	0	50	0	0
	Stage III	0	0	50	0
	Stage IV	2	0	0	48
ANN [all features]		Stage I	Stage II	Stage III	Stage IV
True class	Stage I	50	0	0	0
	Stage II	0	48	2	0
	Stage III	0	0	50	0
	Stage IV	2	0	0	48

**Table 4:** Performance summary of models.

Model	Accuracy (%)	Lowest TPR (%)	Highest FNR (%)
SVM	99.0	96 (Stage IV)	4.0
KNN	99.0	96 (Stage IV)	4.0
ANN	99.0	96 (Stage II, IV)	4.0
Discriminant	99.0	96 (Stage IV)	4.0
Naive Bayes	94.5	88 (Stage III)	12.0
Decision Tree (DT)	91.5	86 (Stage III)	14.0

**Table 5:** Additional performance metrics.

Model	MCC	Cohen’s Kappa
SVM	0.98	0.97
KNN	0.98	0.97
ANN	0.98	0.97
Discriminant	0.97	0.96
Naive Bayes	0.91	0.90
Decision Tree	0.88	0.86

## Data Availability

The original contributions presented in this study are included in the article. Further inquiries can be directed to the corresponding author.
